# Comparison of time-motion analysis of conventional stool culture and the BD MAX™ Enteric Bacterial Panel (EBP)

**DOI:** 10.1186/s12907-015-0010-8

**Published:** 2015-05-28

**Authors:** Joel E. Mortensen, Cindi Ventrola, Sarah Hanna, Adam Walter

**Affiliations:** Department of Laboratory Medicine, Cincinnati Children’s Hospital, MLC1010, 3333 Burnet Ave, 45229 Cincinnati, OH USA; BD Diagnostics, Sparks, MD USA

**Keywords:** Time-motion analysis, BD MAX™, Diarrhea, Bacterial stool pathogens

## Abstract

**Background:**

Conventional bacterial stool culture is one of the more time-consuming tests in a routine clinical microbiology laboratory. In addition, less than 5 % of stool cultures yield positive results. A molecular platform, the BD MAX™ System (BD Diagnostics, Sparks, MD) offers the potential for significantly more rapid results and less hands-on time. Time-motion analysis of the BD MAX Enteric Bacterial Panel (EBP) (BD Diagnostics, Quebec, Canada) on the BD MAX System was compared to conventional stool culture in the microbiology laboratory of a tertiary care pediatric hospital.

**Methods:**

The process impact analysis of time-motion studies of conventional cultures were compared to those of EBP with 86 stool specimens. Sample flow, hands-on time, processing steps, and overall turnaround time were determined and analyzed. Data were obtained and analyzed from both standard operating procedures and direct observation. A regression analysis was performed to ensure consistency of measurements. Time and process measurements started when the specimens were logged into the accessioning area of the microbiology laboratory and were completed when actionable results were generated.

**Results:**

With conventional culture, negative culture results were available from 41:14:27 (hours:minutes:seconds) to 54:17:19; with EBP, positive and negative results were available from 2:28:40 to 3:33:39.

**Conclusions:**

This study supports the suggestion that use of the EBP to detect commonly encountered stool pathogens can result in significant time savings and a shorter time-to-result for patients with acute bacterial diarrhea.

## Background

The World Health Organization has reported that, worldwide, there are nearly 1.7 billion cases of diarrheal disease every year and that diarrheal disease is the second leading cause of death in children under five years old [1, 2]. Each year, diarrhea results in approximately 760,000 preventable deaths of children under the age of five years. Diarrhea in this age group is also a leading cause of malnutrition. Most cases of this disease are related to unsafe drinking-water, inadequate sanitation, and poor hygiene [1, 2].

Detection and identification of the etiological agents of acute bacterial diarrhea are important for both the treatment of individual patients and for the management of diarrheal diseases of public health importance. Conventional bacterial culture remains the gold standard for the aforementioned detection, even though stool culture has relatively low sensitivity and requires a significant amount of labor. The use of nucleic acid amplification methods to detect and identify the etiological agents of acute bacterial diarrhea could have a significant impact on the laboratory diagnostic process, clinical approach, and epidemiology of this disease [3–5].

The objective of this study was to examine the laboratory impact of a new molecular platform (use of the BD MAX Enteric Bacterial Panel on the BD MAX System) on turnaround time, associated laboratory processes, and the cost of providing results with this system compared to conventional culture methods. Results of both conventional culture (including a commercial immunoassay for shiga-toxin) and the EBP, which include tests for the detection of *Salmonella* spp., *Shigella* spp./ Enteroinvasive *Escherichia coli* (EIEC), *Campylobacter* spp. (*jejuni* and *coli*), and Shiga toxin 1 and 2 genes in stool specimens were evaluated. Lean and Six Sigma processes were used to analyze the time from sample receipt to actionable result for conventional stool culture and the EBP. The following “events or decisions per specimen” were determined: any action or thought process that must occur to process and issue a result, the overall distance traveled per sample as a measure of efficiency, and the operating costs of the two systems [6].

(The results of this study were presented, in part, at the 24th European Congress of Clinical Microbiology and Infectious Diseases, Barcelona, Spain, May 10–13, 2014 and at the 114th General Meeting of the American Society for Microbiology, Boston MA, May 17–20, 2014.)

## Methods

Lean and Six Sigma processing analysis were performed to evaluate time-to-results for both culture and EBP testing. By design, this study did not involve human subjects or any patient information. Observations were performed without patient identifiers and additional testing was carried out on discarded, anonymous samples. Any sample ordered for routine stool culture was eligible for inclusion in the study.

### Culture

Clinical stool samples were immediately accessioned and plated upon arrival in the laboratory following standard laboratory practices. They were not stored prior to culture. Sample flow, hands-on time, processing steps, overall turnaround time, and specimen travel distance were measured by two independent observers over the course of three separate observation periods; each observation period was five days. To eliminate any potential of operator-to-operator bias during the study, 11 different laboratory technologists were observed performing all pre-analytical, analytical, and post-analytical culture procedures which occurred at five different laboratory stations: specimen receipt, specimen plating and incubation, culture reading, automated identification (Vitek 2 System, bioMérieux, Marcy l’Etoile, France) and shiga-toxin testing (Immunocard STAT! EHEC, Meridian Bioscience, Cincinnati, Ohio, USA).

In brief, for this study, stool samples that were submitted for routine culture were transported in Cary Blair Transport Medium (Meridian Bioscience, Inc.). Specimens were processed within 2 h of receipt. Initially, samples were inoculated onto the following agar media: 5 % sheep blood, MacConkey, Sorbitol MacConkey, Hektoen, Campy CVA (BD Diagnostics, Sparks, MD, USA). Cultures were incubated under standard conditions. Suspected bacterial pathogens were identified using the Vitek 2 System (bioMerieux) and standard conventional methodologies as needed. Additional testing may have included the following: Salmonella serotyping and Shigella serotyping Becton, Dickinson and Company, Sparks, MD, USA) and Remel RIM E. coli O157:H7 Latex Test (Remel, Lenexa, KS, USA).

Additional data were obtained and analyzed from laboratory Standard Operating Procedures (SOP) used routinely in this particular laboratory. In order to ensure consistency during the study, a regression analysis of measurements, processing, and adherence to the SOP was performed. Correlation studies were performed on independent data sets to ensure no bias was falsely introduced by operator-to-operator performance [7]. The following elements were analyzed: elapsed time, distance traveled, processing steps performed, and clinical decisions, from the time the specimens were logged into the accessioning area until the time actionable results were generated. Processing was observed and data were collected during each of the following notable events: specimen arrival, specimen accessioning, specimen plating and preparation, specimen incubation, first-day plate reading and workup, subsequent day(s) reading, including *Campylobacter* spp. cultures reading, automated identification and additional workup, shiga-toxin broth inoculation, shiga-toxin rapid testing, and verification of results, and entering results into the laboratory/hospital information system.

### EBP testing

Methods similar to those used to evaluate culture processes were measured and analyzed: elapsed time, distance traveled, processing steps, and clinical decisions (also from the time specimens were logged into the accessioning area to the time actionable results were generated). Processing was observed and data were collected during each of the following notable events: specimen arrival, specimen accessioning, control preparation, specimen preparation, instrument preparation, worklist preparation, instrument processing, and result verification. For BD MAX testing, the samples were batched and tested in different batch sizes. Batch sizes ranged from 4 to 24 samples in increments of 4 to mimic routine clinical testing.

### Cost analysis

Standard institutional cost analysis was used to determine the costs for conventional culture and for EBP. The main cost components of the analysis were labor, direct materials and supplies, and general shared costs (Test Site Burden). Hands-on time (minutes) for each step of the cultures and the EBP was multiplied by the average hourly technologist salary/min of labor to determine labor costs. The quantity of each item and the cost of each item used in culture and EBP testing were derived from institutional inventory data. Institutional overhead or Test Site Burden is the cost for basic services such as lights and heat and the cost of common shared laboratory equipment such as incubators, repeat pipettes, etc.

### Institutional review board

It was determined that this study did not meet regulatory criteria for research involving human subjects because the research did not obtain data through intervention or interaction with the individual or identifiable private information. All observations of process were made without any patient identifiers available to the observer. All specimens tested on the BD MAX were anonymous, discarded samples that were only used after clinical testing was completed and for which no patient identifiers were used.

## Results

86 patient specimens were examined. No pathogens under consideration in this study (i.e., *Salmonella, Campylobacter, Shigella* and shiga toxin producing organisms) were detected by culture or BD MAX EBP.

**Fig. 1:**
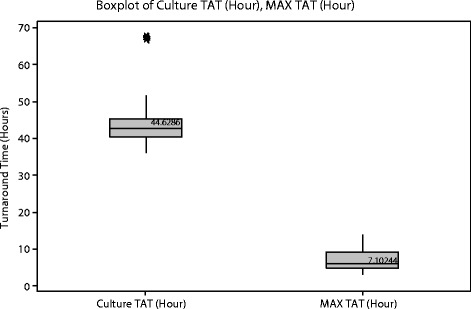
The mean turnaround time (TAT) to reportable results for 86 routine stool cultures and 84 samples tested with BD MAX EBP. Legend. *Represents 4 outlying culture results that required additional testing for confirmation of the results. All final culture results were negative for pathogens

To enable a comparison, simultaneously, 84 alternate specimens tested by EBP were processed across six batches of differing size (4 to 24 samples each) on the BD MAX platform. Processing and turnaround times of routine cultures were compared to the process and turnaround times of EBP testing. The mean time to reportable result for 86 routine cultures was 44:37:00 (hours:minutes:seconds) (+/− 8 h, 10 min) (Fig. 1). If potential pathogens were detected that required additional testing, the time to final result ranged from 97:18:17 to 145:27:11.

Although the time to perform EBP testing is approximately two hours, EBP is designed and best used to batch specimen testing at reasonable intervals as determined by each laboratory. If a reasonable operational model is two EBP runs per day (one in the morning, one in the afternoon), the time to reportable EBP results would be, at most, 07:06:00 (no standard deviation; all variability dependent upon batch size and timing of run). Hands-on time per specimen was 0:01:30 (+/− 19 s). With an assumption of two EBP runs per day and 90 s hands-on time/specimen, there was an 85 % reduction of time to reportable results compared to culture.

### Process Steps for Culture and EBP

**Fig. 2:**
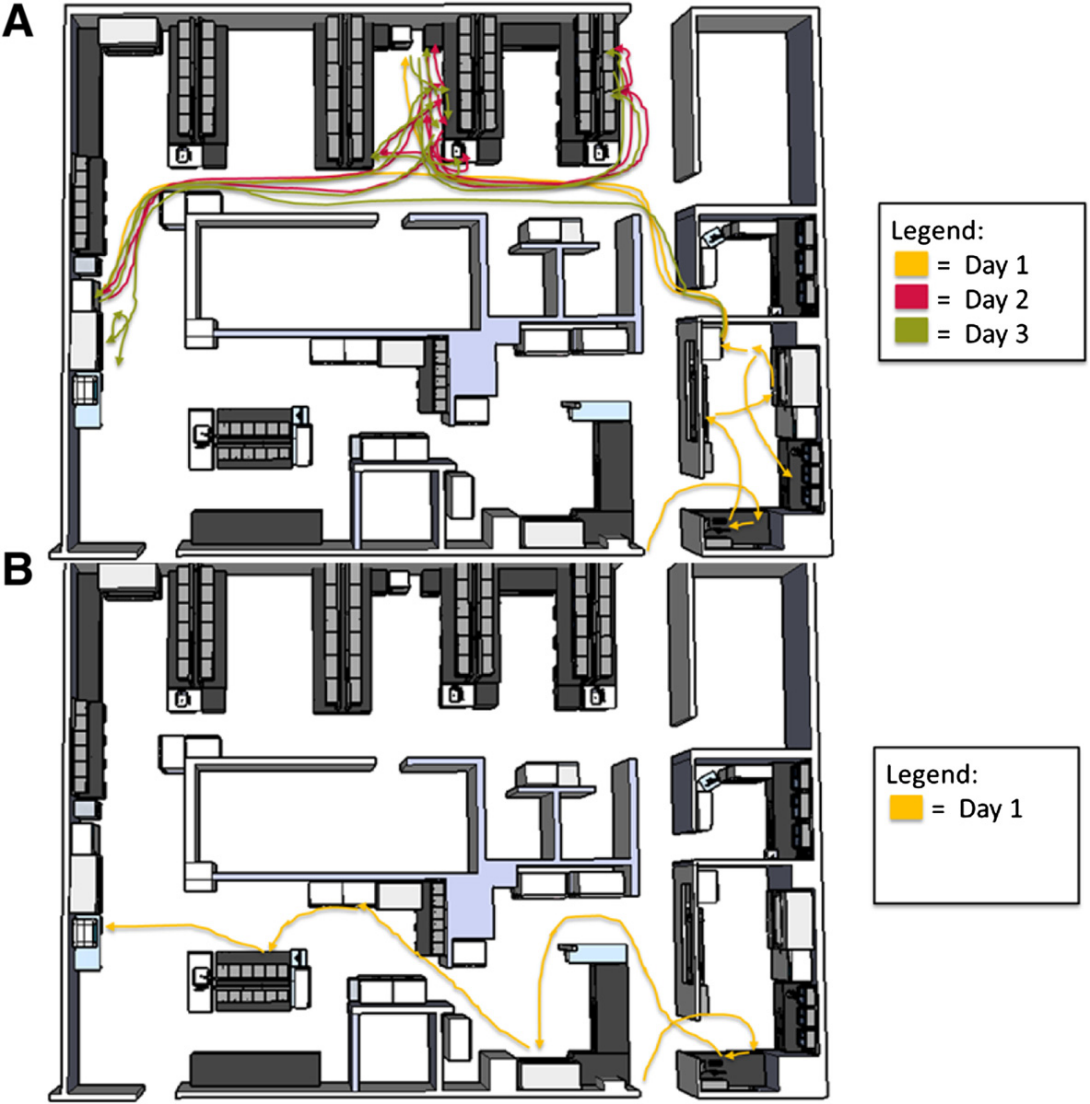
Spaghetti Diagram of Process Flow for Routine Stool Culture (**a**) and BD MAX EBP Testing (**b**)

Technologists made an average of 141 and 25 decisions per culture and EBP test, respectively. Thus, EBP testing required 82 % fewer decisions than did culture (Table 1). The number of steps and processes in each unique laboratory can be represented by a spaghetti diagram of process flow for culture and for EBP testing (Fig. 2).

**Table 1:** Average Number of Process Steps (Decisions/Manipulations) Involved in Routine Culture and BD MAX EBP Testing

Process Steps	Routine Culture	EBP
Receipt	3	3
Accession	7	1
Routine culture		
Blood agar	43	-
MacConkey agar	26	-
Hektoen	26	-
Sorbitol MacConkey	14	-
Shiga toxin testing	4	-
Sample Preparation	-	8
System operation	-	13
Total activities	141	25

### Cost analysis

Detailed costs are listed in Table 2. The basic labor to process and handle a stool culture was 0:15:00 – 0:17:00. Approximately 20 % of the cultures required additional process steps to rule out potential pathogens; these additional steps resulted in additional labor (0:35:00 – 0:40:00) and supplies. The EBP required 0:01:28 hands on time.

**Table 2:** Cost Analysis of Routine Stool Cultures and BD MAX EBP Testing

	Stool Culture			EBP		
	Cost/Unit	#Units	Cost	Cost/Unit	#Units	Cost
**Basic test**						
Labor -Technologist time (minutes)	0.45	15 - 17	6.75 – 7.65	0.45	1.48	0.67
Information System labels	0.05	3	0.15	0.05	2	0.1
5 % Sheep Blood agar plate	0.25	1	0.25			
MacConkey agar plate	0.25	1	0.25			
Campy agar plate and BioBag	2.39	1	2.39			
Hektoen Enteric agar plate	0.31	1	0.31			
MacConkey Sorbitol agar plate	0.44	1	0.44			
Shiga toxin test kit and MacConkey Broth	13.52	1	13.52			
Disposable 10 μl loop				0.02	1	0.02
Enteric Panel Kit				30 – 35.00	1	30 – 35.00
MAX test cartridge				0.40 – 0.65	1	0.40 – 0.65
Test site burden	1.00	2	2.00	1.00	1	1.00
Additional workup*						
Labor -Technologist time (minutes)	0.45	35-40	15.75 – 18.00			
5 % Sheep Blood agar plate	0.25	1-3	0.25 – 0.75			
Vitek-Gram negative ID card	6.00	1-3	6.00 – 18.00			
RIM EC O157:H7 test	0.59	1	0.59			
**Total Cost (in $)**			$26.06 – 64.30			$32.19–37.44

*20 % of cultures required additional labor and supplies to rule out potential pathogens

## Discussion

A number of molecular platforms have been evaluated for specific specimen types, including stool, and potential pathogens in clinical laboratory settings and have been shown to be highly sensitive and specific when compared to conventional methods [3–5]. The BD MAX platform has been evaluated for the detection of MRSA and more recently stool pathogens [8, 9]. Recently, several investigators have recognized that beyond scientific validation, these platforms need to be evaluated for their impact on the operations and the time to reportable results in clinical laboratories [7, 10–12].

One of the more challenging parts of this study was accounting for all of the costs. The lack of positive samples with target stool pathogens did not allow a complete determination of the costs and labor associated with routine stool cultures. A community outbreak of acute bacterial diarrhea might significantly impact both labor and supplies for both of the methods in this study. In addition, it was difficult to account for the individual variability between technologists in the workup of stool cultures. Differences in individual technologists and their experience could have affected the extent of work and supplies needed for a culture. Nonetheless, including multiple technologists in the performance of this study more accurately represents real-world performance of the two methods than, for example, performing the study with specified research technologists. The use of a significant amount of shared equipment for routine cultures makes complete accounting for the portion of the cost of equipment such as water baths, incubators, storage rack, microscopes, etc. assigned to each culture difficult. At our institution, we use the somewhat arbitrary “Test Site Burden” as one mechanism of sharing these costs. An additional impact within the laboratory is the shift in supplies storage. A significant number of different media and tests are need for routine cultures and most of these require refrigerated storage. A move to the EBP assay would reduce the number of tests and the amount of media. In addition, adoption of the system would shift much of that storage to room temperature.

In contrast to culture, the cost of operating the BD MAX was more easily captured. However, there are several additional issues that affect the cost of operating the EBP assay that a clinical laboratory would need to consider. Because of how the disposables are constructed, samples can be run in various size batches. To optimize and reduce cost, the ideal batch size is 24. To optimize and reduce turnaround time, the ideal batch size is as small as possible. Use of fewer stools samples per batch would have a minor effect on the cost of the test. The cost of the EBP would not change with a positive result; however, a positive EBP would require a follow-up culture to allow serotyping, antimicrobial susceptibility testing as appropriate, and epidemiological studies, including time and supplies to send isolates to the State Public Health Laboratory. Depreciation of instruments is an important consideration if the instrumentation is purchased outright. Our analysis did not include the cost of the analyzer as that cost per assay is directly related to the volume of assays performed on the instrument. Finally, the cost of service contracts is often not considered as part of the cost of a test, but can represent a significant cost to the operations of the laboratory.

As clinical microbiology laboratories move from traditional culture based methods to instrument based molecular methods, we need to look carefully at scientific merits of the various options, but we need also to look at the turnaround time of results, associated laboratory processes, and the cost of providing results with this system compared to conventional culture methods in our laboratories.

## Conclusion

This study supports the suggestion that use of the BD MAX EBP can save significant time (over that required by culture) in the laboratory diagnosis of acute bacterial diarrhea caused by *Salmonella* spp., *Shigella* spp./(EIEC), *Campylobacter* spp. (*jejuni* and *coli*), and Shiga toxin producing *E. coli* which are responsible for 95 % of acute bacterial gastroenteritis. The use of a flexible and focused approach to identifying enteric pathogens (bacteria, viruses & parasites) based on patient history or risk, clinical presentation or clinician’s preference is aligned with widely recommended clinical algorithms which not only potentially streamline laboratory testing and workflow in a cost effective manner, but also provide physicians with timely results which improve the standard of care for common causes of gastroenteritis. As additional nucleic amplification assays become available, the impact of the use of focused versus comprehensive panels will continue to be evaluated for their respective clinical relevance, cost and work flow implications.

## Abbreviations

EBP: Enteric bacterial panel
EIEC: Enteroinvasive *Escherichia coli*
SOP: Standard operating procedures
MRSA: Methicillin resistant *Staphylococcus aureus*
